# Treatment of Abscess

**Published:** 1861-11

**Authors:** 


### TREATMENT OF ABSCESS.

It has been suggested that in the treatment of alveolar abscess, the persulphate of iron may be employed with very desirable results; being used in the same manner as creosote for the same purpose. The channel is opened freely with the lancet to the bottom, and all extraneous substance removed by washing, swabbing, etc., and then painting the entire inner surface with the sulphate. This cauterizes the surface, and forms coagula soon. After this healthy sloughing takes place and granulations spring up and the opening is soon closed. This remedy is worthy of trial. T.

